# On Excessive Trust in Nature in the Cure of Diseases

**Published:** 1846-10

**Authors:** J. A. Symonds

**Affiliations:** Consulting Physician to the General Hospital, Bristol


### PART THIRD.

### CONTRIBUTIONS TOWARDS THE ADVANCEMENT OF THE

### Natural History and Treatment of Diseases.

---

### I. ON EXCESSIVE TRUST IN NATURE IN THE CURE OF DISEASES.

BY J. A. SYMONDS, M.D.,

Consulting Physician to the General Hospital, Bristol.

(*In a Letter to Dr. Forbes.*)

Bristol, August 20, 1846.

My dear Sir,—I was much gratified by your remarks in the Journal for April, obviously intended to counteract some of the impressions which had been produced by your article in the January Number. I knew you to be a vigorous eclectic in practice, but I feared that the vehemence of your protest against the manner in which the *medicina perturbatrix* is sometimes exercised, and that the pains which you took to hint or assert the adequacy of Nature alone to the cure of many and very important diseases, might be prejudicial in two ways. In the first place, I apprehended that your frank and plenary confessions as to the inutility or injuriousness of some of the ordinary methods of cure might be caught up, not only by ignorant and pestilent empirics, but also by the fanatical apostles and disciples of the homœopathic and hydropathic heresies; and that the confidence of the laity in legitimate medicine might be thereby shaken more than it has been already. This *læsa fides* is no small addition to the difficulties and perplexities which, under the most favorable circumstances, must often beset the honest and scientific practitioner. On the other hand, I was fearful that your recommendation of a trial of the undisturbed play of the *vis medicatrix* might induce in some minds a general indolence of practice, and in others might encourage that liability to paralysis of judgment, which, by the bedside, has often produced absolutely fatal results, both to the patient and the practitioner. I know that you maintain that there are many cases in which, with our present knowledge, it is our bounden duty to exercise great vigour in our interference with disease. We must act up to our lights. Be they strong or be they feeble, we must still act up to them. The *laissez-faire* system is good, in so far as it gives free scope to healthful energies; but, if it presumes to tolerate the powers of darkness, the sooner it goes to Tophet, the better for poor man, who has hard work enough as it is in fencing off disease and the devil.

I repeat, then, that it gave me great satisfaction to find that you had so distinctly expressed your belief in the necessity of an active interference with disease, and in the efficacy of many of the received remedies, and that you wished to be understood as having only entered a strong caveat against the blind heroics and unreasonable polypharmacy still too prevalent, and as having suggested a more thorough and searching inquiry into the natural history of morbid phenomena, and into the principles upon which therapeutics should be founded.

Your essay elicited some very interesting correspondence—Dr. Laycock's paper must have been read with the same satisfaction as the many other contributions which have been bestowed by that enlightened physician on the philosophy of medicine. The letter to which the honoured name of Dr. Andrew Combe is attached, and the anonymous epistles in your last Number, appear to me to set rather too much in the direction of the expectant method. You will therefore, perhaps, excuse me, if, at the risk of uttering things trite and tedious, I venture to make a few remarks, the drift of which will be opposed to the non-coercive system of treating diseases.

The question before us is, whether it is safer to leave diseases to their own course, or to interpose what we call remedies? And really this is a very frightful question to be asking at the present date of the world's history, when medicine has been proposed as an art two thousand years and more! What! is it a probability admitting of debate, that, of the countless swarms of ghosts that have passed "the melancholy flood," medicine has introduced to the "grim ferryman" those whom kind Nature would have held back? But we need not frighten the imagination with horrible surmises of forthcoming corollaries from a possible conclusion, which, if it were true, would be one of the mournfullest truths in the universe.

I confess that Dr. Fleischmann's table somewhat startled me—for a moment it seemed to be the most awful statement in numbers that I had ever met with. I say awful, because, if received as it stands, it would require us to revolutionize from the foundations the whole constitution of medical art. To think of 100 recoveries out of 105 cases of peritonitis!—with no treatment but what was dietetical, for I entirely agree with you in computing the globules as = 0. What sort of disease peritonitis may be in Austria, I have no knowledge from experience; but, if this statement be correct, I have no hesitation in expressing my belief, that it must be a different malady from that which is associated in my mind with the name. But, on further examination, I noticed anomalies which soon removed the discomfort which the first view of the table occasioned. There appear to have been only six cases of enteritis. This may perhaps be accounted for by a restriction of the name to inflammation of the muscular coat of the intestine. There is no mention of enteritis as seated in the mucous membrane, and I therefore infer that it is included under diarrhea. Yet, strange to say, this last-named category numbers only 114 cases—that is, only 9 more than peritonitis. Again, while 300 are assigned to pneumonia, bronchitis has only 15! At first it seemed probable that the milder cases of disease are not admitted into the hospital; but, were this the case, how are we to explain 300 affixed to tonsillitis? *Gout* appears to be common among the paupers of Vienna;—possibly the paternal government makes them luxurious. There are no less than 102 cases—more than half the number ranged under the head of rheumatism (188). These *peculiarities* of Dr. Fleischmann's table are, I think, sufficient to justify us in excluding it from the present inquiry.

Whether a true art of medicine does or does not exist, it must be allowed that there *ought* to be one. Man has succeeded in conquering or repressing so many of the adverse powers in Nature, and has subjugated so many others to his purposes, that we might by analogy expect him to acquire some dominion over the *vis vitiatrix* and the *vis necatrix*. The existence of such forces in Nature is apt to be forgotten by physicians, who, from the influence of the old Hippocratic doctrines, got into the habit of representing Nature, φυσις, as all-beneficent—as if she had no poisons, generated no diseases, and allowed nobody to die if she could help it. Now, it would be contrary to all other experience under the sun, if it should be discovered that men of genius and patient observation have for two thousand years been watching a certain set of phenomena with a view to altering them and making them serve their purposes, and have tried a number of experiments to that effect, and yet have accomplished nothing, or next to nothing! Let it not be said that, though men have been so successful with the mechanical and the fine arts, like results were not to be expected when they had to deal with *vital* phenomena. Has nothing been done in agriculture, horticulture, and the breeding of animals, &c. &c.? Art, after all, is but Nature in a new form—a fresh arrangement of the forces of Nature, compelling them to work under new conditions.

Much of the practice in the ordinary system of medicine is a strict imitation of salutary processes in Nature, and its object is to make such processes supersede the *baneful practice of Nature*, who, though she knits up wounds with her adhesive inflammation, by the very same method glues the intestines into fatal entanglements, shackles the heart, and chokes up the windpipe. In *this* man she soothes "the grief of a wound" by pouring out serum; in *that* she makes the same effusion effectually close the rima glottidis. She spirts blood from the hemorrhoidal vessels of Paul, who blesses her for saving him from apoplexy; of which very disease poor Peter dies, because she has poured the same fluid into his lateral ventricles. And so on, for man's body is a microcosm in which one sees the play of Zoroaster's antagonist principles. Nature is ever the same. Blessings are mixed with curses—the poisonous berry and the nutritious root are found in the same plant. There are balmy dews and pestilent fogs—fertilizing streams and destroying deluges—and the lair of the murderous lightning is in the cloud that floats across the blessed sunshine. But man is sent into the world to wage eternal war with the evil one—and woe betide him, if he lets the demons of disease get an advantage over the angels of reparation, for want of his friendly and judicious interference.

I think, then, that there *ought*, in conformity with man's other achievements, to be an art of medicine. And if it can be proved in any case that human skill has successfully interposed where Nature would assuredly have failed, we have good reason for caution against a general trust in the capabilities of Nature, and a general distrust of those of art. Let us call to mind two or three instances of Art versus Nature. Surgery suggests, among a host of other triumphs, the operation for strangulated hernia, ligatures on arteries in aneurisms and hemorrhages. A man's life is ebbing: he cannot tell why. He looks bloodless—he knows of no hemorrhage—there is no apparent disease. The physician insists on his using a commode for one day instead of a dark water-closet. Florid arterial blood is observed in the evacuations, and, on examination of the rectum, a painless bleeding tubercle is found just within the verge of the anus. The aid of a surgeon is requested for the application of caustic or a ligature, and the patient takes out a new lease of existence. In obstetrics what wonders are wrought by active interference with Nature, e.g. in placenta prævia, arm presentations, retained placenta,—nay, craniotomy.

Look at medical treatment in the eye. To say nothing of extracting cataract and making artificial pupils, who has not seen cases in which Nature had been doing nothing, or worse than nothing for months, and yet, on a happy application of some positive disturbing agent, no doubtful invisible medication, but such a thing as lunar caustic or blue-stone, the disease is gone. Or take the skin. A lady suffers from an obstinate lepra—she has long given up medicines—she has been content to mitigate the disease by diet, and baths, and regimen—she has had the disease for years. She is moderately bled—an issue is inserted in the arm, and she takes that triple poison, the hydriodate of arsenic and mercury: in six weeks she is cured. I have chosen for examples, diseases the evidence of which is objective. Neuralgia and other subjective phenomena are open to more doubt.

But to come to the cases in which it is especially needful to have our minds made up as to the right course of practice—I mean the acute visceral inflammations—when I become satisfied that these diseases may be left to pursue their own way, my belief will have suffered a more remarkable *bouleversement* than it has ever yet undergone. We know well enough what these diseases are capable of doing in the way of disorganization, whether meddled with or not. We have met them in the latter predicament in the neglected cases presented to us in dispensary and hospital patients; also in cases of latent inflammation, revealed only by the scalpel—cases in which *Nature* had been stealthily prosecuting her deadly work. But it might, perhaps, be said that there is no sufficient evidence to prove that such cases would have had a better issue had they been placed under orthodox treatment. Some years ago I was called to a patient who had been ill four days. He had been seized suddenly —the symptoms were those of peritonitis. For reasons which it is needless to state, not a single active measure had been employed. He had been confined to bed, taken diluents, and very trifling medicines. When I saw him the belly was tympanitic, with complete constipation and sickness. The pain had lessened, but there was tenderness; the voice had become husky; there was full evidence that the usual effusions had occurred in the peritoneum. I thought the case all but hopeless, but I practised some moderate depletion. Some alleviation ensued, but nothing more. He was not laid prostrate by the remedies, and, as a proof of it, he lived to the seventh day from the attack. He was a strong healthy man of 50. The necropsy showed the extensive adhesions, &c., of sthenic plastic peritonitis. Do you doubt that if, at the onset, the patient had been bled, leeched, and taken opium freely (with or without calomel), he would have had a far better chance of recovery? I have said, "we must act up to our lights." Now, one has repeatedly seen cases begin in a way closely similar, and after, or because of certain remedies, yielding, as to the most essential points, in two or three days—nay, in a day;— the inflammation stopping before fibrin has been effused. I don't believe there can be a greater sceptic in these matters than I am; yet even my Pyrrhonism is overcome when I see the marvellous change induced at the *onset* of an inflammation by a bleeding, a poultice, and an opiate. Even if it be maintained that the *symptoms* only are abated, e. g. the pain and tenderness, and the vomiting and constipation, and the rapid pulse, and hot skin, and scanty secretion, but that still the essential disease, the pathological lesion, the *causa causans*, the *vitium vitians*, goes on a certain course, and stops only when Nature thinks proper—still I say, thank God for the lancet and opium! My patient is lapped in balmy slumber, and I am quite at ease as to the issue of the malady.

Take another instance. I was called up in the night to a lady, who I found had been ill several days. She had been treated with very mild remedies for what appeared to be hepatic congestion. She was very feverish, and breathed in a way that gave me the impression, when I first set eyes on her, that she was suffering from pneumonia; but I listened to the chest, and failed to find any sign of inflammation within the thorax. The pulse had an irritable character, and there was slight delirium; and, in the absence of proof of local disease, it was decided to try an expectant method, on the probability of the case turning out to be one of fever. The next evening things were rather worse. Again I explored the chest, and this time listened where I *ought* to have listened before, but where I had been deterred by a blister, which had been placed over the region of the liver. There I found crepitation and bronchial respiration. The case was clear. It had been occult pneumonia, which had possibly begun in a part more removed from the surface. Then came a terrible antiphlogistic visitation; all the great guns were fired: the patient recovered perfectly. Relief and abatement of disease ensued at once; but a check was not at once given to the hepatization, which involved two thirds of the lung. The most decided improvement in the signs, both physical and rational, was coincident with mercurial stomatitis. I do not deny that I have known hepatization consequent on neglected pneumonia get well without active treatment; but it has been in a prodigiously longer time than when antiphlogistic remedies had been employed.

Bear with me, if I narrate one more case. A young lady had been ill for many months. For several weeks she had been under a homœopathic practitioner, who was, of course, treating her symptomatically. She got worse, and ordinary medicine was again appealed to. We found an enormous collection of fluid in the left pleura, pushing the heart far over to the right side. We practised very moderate depletion, chiefly with a view to favour absorption; blistered largely; and exhibited diuretics. The case is now under treatment; and, as I have repeatedly seen in like cases, the fluid has been traced receding in proportion as the excretory organs have acted freely, and the discharge has continued from the blistered surface. The heart has resumed its proper habitat, and the symptoms (especially the dyspnœa) are greatly mitigated; but the case will probably end in phthisis.

If one were at a loss for instances in which art interfered with advantage, when Nature was doing nothing, or rather doing mischief, I doubt if any instance could be more striking than the cure and relief of many of the diseases of children by lancing the gums. On the expectant method a child may, after many days of suffering, and after incurring perils in various organs, at last recover when the tooth has pushed its way into the light; but all the pain and jeopardy might have been spared by one or two free incisions.

The efficacy of medical treatment is, I think, evidenced by the short time requisite for the cure of diseases, which, cured by Nature, occupy a far longer period. Within the last six months I have watched two cases of subacute pleuritis, both well marked by the auscultatory signs. In both there were reasons, from the peculiar constitutions of the patients, for avoiding general blood-letting and the stronger medicaments. They both recovered; but I have no doubt, from my experience in similar cases, that a more active treatment, not violent, would have reduced the disease in at least half the time. Whether it would have been ultimately a *gain* might be questioned; but such instances show the power of art over morbid processes.

Time does not allow me to touch on the *reasonableness* of the antiphlogistic treatment—as, for examples, the influence of venesection on the action of the heart, arteries, and capillaries, to say nothing of the blood; the operation of opium on the neurotic element of inflammation—of mercury and antimony on the excretory actions; and the close correspondence between the effects of such agents and the processes which appear to be instrumental in resolving diseases which are cured spontaneously.

But what shall we say of fevers and the exanthemata? Only what nine tenths of eclectics would affirm, viz. that the less heroically they are treated the better. And why? Because neither theoretical nor empirical therapeutics have suggested remedies that appear to tell on the essential part of the disease, that is, the poison in the blood. At least, I know of none, unless it be chlorine in scarlatina and typhus, when accompanied with putrid symptoms. Yet we cannot but hope that, when the pathology of the blood has reached a development approaching to that of the tissues, we shall have remedies equally direct and appropriate.

I am not fond of arguments from final causes; but can it be doubted that the various medicines we possess were, as such, a part of the plan cf the universe, designed to have relation to morbid states of living organisms, as much as esculent matters to healthy conditions? What! were diseases planned? It would seem so; and that it was no less needful that men should depart from the stage of life than they should enter upon it. Disease and death are as *natural* as health and life. And lest it should be objected to this view, that if diseases were intended to sweep men from the earth, antagonists would not be provided for them in the form of medicines, I may remark that this is only one of a multitude of illustrations of the fact that it has pleased Divine Providence to order the world upon a system of checks and counter-checks. Few things show this more strongly than the predatory instincts of one set of animals, and the provisions in a weaker set for eluding and guarding against the attacks of the former.

In your pamphlet, page 31, some excellent reasons are adduced for believing that "Nature can cure diseases without the assistance of art;" but they appear to me to admit of some limitation. Thus it is quite true that diseases are cured "among uncivilized nations, of ancient and modern times, under the sole influence of magic, charms, or other practices equally ineffective;" but, on the other hand, in proof of the inefficiency not only of these methods, but also of Nature herself, one might appeal to the eagerness with which savage tribes invite the medical aid of travellers, and to the extravagant confidence they repose in it, as compared with their anticipations of benefit from their own remedies.

With regard to the expectant system of medicine, though it became very prevalent, we must not forget that its failures generated that disposition to violent heroics which characterised the practice of the first quarter of the nineteenth century. Should the present overweening trust in Nature continue, we may apprehend a similar reaction.

The apparent success of quack medicines, inferred from the large consumption of them, does, I agree with you in thinking, in some measure prove that a vast number of diseases get well spontaneously. Yet we may err in presuming that the popularity of such remedies depends mainly on their seeming utility. Much of the estimation in which they are held may, I suspect, be assigned to the circumstance that a large number of them are, more or less, derived from the class of purgatives; the action of which is always in high favour with the laity. Many a physician has heard the reproaches of patients for the inertness of his prescriptions, simply because they had not produced catharsis—a process associated in their minds with anything like activity of treatment.

As for hydropathy, the subjects of its milder processes may, perhaps, fairly be said to owe their recovery to the *vis medicatrix;* but in its full administration the system is a glaring instance of *medicina perturbatrix*. It is drugging, and very dangerously, with aqua pura.

That practitioners, as they advance in life, become more and more inclined to confide in the resources of Nature, would imply that experience has not taught them to attach as much value to artificial methods of cure as at earlier periods of their career. But we must not lose sight of the fact that age brings not only wisdom, but also an indisposition to enterprise in our own as in other professions. There is also another consideration to be borne in mind. Physicians, whose practice is chiefly consulting, meet with a large proportion of chronic cases; and, as Dr. Alison has judiciously remarked in his essay on Inflammation, in the 'Library of Medicine,' with reference to the value of blood-letting, they are often called in, either when the time has gone by for the remedy to be either safe or advantageous, or when it has already been practised as far as the patient's strength would allow. They are, therefore, less likely to be impressed with its efficacy than those who are present at the onset of the disease, and see it quelled at once by the prompt administration of this measure.

I have not adverted to hygienic methods, which are said to be capable of curing diseases without the aid of other remedies. Allowing this to be true, we must, nevertheless, perceive that they are interpositions of art. If a patient is confined to bed, when but for orders he would be sitting up or walking about; if he is restricted to bread and water, or milk, when otherwise he would be living as usual; if a particular temperature of his room is maintained, &c. &c., and he gets well—his case, surely, ought not to be adduced as an example of the curative powers of Nature. There has been a decided interference of Art.

I should deeply regret were these cursory remarks to give you the impression that I underrate the reparative powers of Nature, or that I advocate anything approaching to *meddlesome* practice. My intention was to hint, first, that *à priori* we might expect there should be an art of medicine—an art analogous to all other arts; for they all consist in the subjugation of the materials and forces in Nature to the wishes of man; and, secondly, that it is an error to speak of salutary changes, as those of Nature *par excellence*, for she is destructive as well as conservative; exciting processes which tend at one time to disorganization and death, at another to reparation and life; and that it is needful to watch her actions with rigorous attention, nay, with suspicious scrutiny, and to do our best towards encouraging the *vis medicatrix*. He who has learned neither to wait too long, nor to interfere too soon, has made an attainment scarcely inferior to that of knowing *what* remedy should be employed.

I have carefully read your cogitanda; but though there is scarcely a suggestion with which I do not willingly accord, I am of opinion that it is not so much a revolution or reformation of medicine that is wanted, as a more thorough and extensive education of practitioners in the art and science of medicine as it at present stands. The most intelligent and accomplished must sigh for more knowledge in a thousand directions, but the interests of medicine very loudly demand that its professors should *know* what is already *known*. Were pathology pursued and applied, and the operation of remedies observed and made use of, after the methods and rules laid down and illustrated by Dr. Williams, in his 'Principles of Medicine,' and by Dr. Watson, in his 'Lectures on Practice' (I instance these works as *typical* in their respective departments), both the profession and the laity would have practical proofs of improvement in our art, sufficient, I think, to satisfy them that knowledge has been sought in the right tracks. "Our venerable mother," *me judice*, is neither sick nor sorry; nor does she, by reason of age or decrepitude, require a Medea's cauldron in order to come out as "Young Physic." In fact, she is not old enough; she is undergoing development, advancing *towards* maturity, though slowly, and a long way off the goal.

The desiderata are many and great. Not the least is a knowledge of the *natural history* of diseases left to themselves. But who shall furnish this? No one can conscientiously seek it in cases which experience has taught him are capable of arrest or abbreviation by art. Neither you nor I could make up our minds to stand composedly by the bedside of a patient suffering an acute inflammation of the pleura or peritoneum, and in the characters of scientific umpires to say to the sanative and morbific forces, "Now, you antagonist *vires*, we are here to see fair play, a clear stage, and no favour. Our part is not to interfere in your deadly struggle, but to note your relative prowess—*laissez-aller!* Philosophic eyes are on you; and God defend the right!" But as we are not prepared for this position, you ingeniously suggest that we should turn to account the records of homœopathic medicine, as containing reports of maladies that have pursued their course unaltered by remedies. But I fear it is impossible that a physician, under the influence of Hahnemannian doctrines, should observe as *we* should observe; for even minds the most exempt from the bias of preconceived views cannot help, in their observations, seeing more or less the reflex of themselves. An observation is a compound result of the thing observed and the observing mind, the latter being frequently the predominating element. A homœopathic mind could hardly give the same copy of a pathological phenomenon as one that had never succeeded in reconciling itself to the ineffable absurdity involved in the doctrine of infinitesimal doses. The conclusive *reductio ad absurdum*, effected in your article, and in the essay of Dr. Alexander Wood, compels one to suppose that those who have received the doctrine in question have done so in accordance with that strange paradox of Tertullian, "Credo quia impossibile est;" while to us it is a great trial of catholic feeling to forbear exclaiming, when such insults are offered to our reason, "Incredulus odi!" Were other arguments against this fantastical system, with its fractional truth of "similia similibus curantur," and its delectable *psoric* figment, wanted, I think a sufficient one might be found in the improbability that a therapeutical system has a sure foundation, which might be practised quite as well by a person whose knowledge of disease is purely symptomatological, as by one who is profoundly versed in modern pathology. The knowledge which is made up of the coarse or spider-web speculations of other men's minds (and a great deal of medical learning, we may concede, belongs to such a category) may come to nought; but that which is derived from a careful and enlightened observation of natural processes, in health and disease, must sooner or later be susceptible of practical application. But homœopathy has no place for the results of the scalpel, the microscope, and the test-tube.

It has been urged by one very estimable writer, that homœopathy ought to have a fair experimental trial, and that it is unjust to reject the system on *à priori* evidence. But to this I would answer, that life is not long enough for making experiments, unless they are suggested by some antecedent probability. Because a dreamer or dupe tells us that he has a method for extracting sunbeams from cucumbers, are we, in order to show our freedom from prejudice, to waste precious time in putting his nonsensical process to the proof? And because we do not choose to steal the time due to more promising pursuits, are we to be everlastingly reminded of the infamy attached to those who opposed the discoveries of Harvey and Jenner? If, on every occasion that these immortal names have been invoked, a real discovery had been announced to the world, of only one hundredth part of the value which belongs to the doctrine of the circulation and to vaccination, few mortals would become weary of life, and "age, ache, penury, and imprisonment," would become unknown evils. But stars of such magnitude are not born every night. Long ages of darkness must overshadow the world between such portentous births. And yet, if an enthusiast takes it into his head to walk about with a farthing candle, and fancy it "light from Heaven," we are not allowed to smile at his delusion till we have climbed up an observatory designed for watching the very stars, and from so exalted a position taken note of so ridiculous a phenomenon!

Dr. Andrew Combe tells us that we must carefully distinguish the principle of "*similia similibus*" from that of the "infinitesimal doses;" and he suggests that we should test the former separately. But to do this, we must begin an entirely new line of observations. And what is to induce us to administer a scruple of jalap for a looseness, and a grain or two of opium for a lethargy? I suppose Dr. Combe would say, the testimony of homœopathic practitioners. But the observations of these gentlemen on the "like-by-like" doctrine are inextricably mixed up with the alleged effects of infinitesimal doses, and are therefore quite useless to the proposed investigation. Doctor Minimus may aver that he has cured gastritis by decillionths of arsenic, but is Doctor Medius warranted by this testimony in trying to do the same with sixteenths or twentieths? Certainly not. The records of homœopathic medicine give no help to us in solving the question of the sufficiency of the "*similia similibus*" theory separately from the minute doses. Then what *is* to show us the duty of departing from orthodox practice? Is it the statement that Hahnemann persuaded himself that he had seen ague brought on by taking bark? *Quousque tandem?*

O, prophet-sage of Cos! well might you mourn over the brevity of life and the slow progress of art, for you doubtless foresaw that, after more than two thousand years, physicians would fritter away the one and impede the other, in proving or refuting the notions of so whimsical a brain as Samuel Hahnemann's!

But I am myself encroaching on this short life with this long letter; and I fear that after all I have been emphasizing truisms.

Yours, very faithfully,

J. A. Symonds.

